# Behavioral Flexibility and the Conservation Value of Howler Monkey Populations in Small Habitat Patches

**DOI:** 10.1002/ajp.70182

**Published:** 2026-06-23

**Authors:** Sebastián Bustamante‐Manrique, Vinícius Klain, Júlio César Bicca‐Marques

**Affiliations:** ^1^ Laboratório de Primatologia, Escola de Ciências da Saúde e da Vida Pontifícia Universidade Católica do Rio Grande do Sul, PUCRS Porto Alegre Brazil; ^2^ Programa de Pós‐Graduação em Ecologia e Conservação da Biodiversidade, Laboratório de Ecologia Aplicada à Conservação Universidade Estadual de Santa Cruz, UESC Ilhéus Brazil; ^3^ Centro de Biotecnologia, Departamento de Biologia Molecular e Biotecnologia Universidade Federal do Rio Grande do Sul, UFRGS Porto Alegre Brazil

**Keywords:** activity budget, conservation, ecological flexibility, feeding ecology, home range, use of space

## Abstract

Species occupying small habitat patches and exhibiting behavioral and ecological flexibility are more likely to persist in fragmented landscapes. However, when isolated populations are not self‐sustaining in the long term, translocation may be required, making an understanding of their behavior critical. Howler monkeys (*Alouatta* spp.) show high tolerance to habitat restriction, which has been linked to a highly folivorous diet and the exploitation of non‐tree plant and non‐native species as food sources. We assessed how the size of the habitat patch influences home range size, day range, time spent feeding, young leaf and fruit consumption, and diet richness, the relationships between these behaviors, and the effect of group size and study length on them using Generalized Linear Mixed Models and full model averaging based on a dataset of 97 studies conducted at 62 locations on the behavior of 124 groups representing nine species. Habitat patch size predicted home range size, which showed a positive effect on fruit consumption. Diet richness increased with increasing mean day range. No variable predicted day range, feeding time, and young leaf consumption. The limited and uncertain predictive power of habitat patch size for most variables reflect the unpredictability of the behavioral and ecological responses of howler monkeys to varying conditions of habitat quality. Our findings are consistent with the possibility that the behavioral flexibility enabling howler groups to survive in small, isolated, low quality habitat patches could enhance their value in metapopulation management strategies aimed at promoting long‐term persistence of the species in human‐modified landscapes.

## Summary

- Habitat patch size predicts home range size in howler monkeys.
- Fruit consumption increases with home range size.
- Dietary plant species richness increases with mean day range.

## 1 Introduction

The concept of a tolerance niche, proposed in the context of climate change, describes environmental conditions under which individuals can survive, but populations fail to achieve long‐term demographic viability (Sax et al. 2013). In anthropogenic landscapes, habitat patches occupied by wildlife may therefore represent tolerated rather than truly suitable environments, effectively functioning as population sinks (Furrer and Pasinelli 2016). This perspective is particularly relevant for small, isolated populations vulnerable to inbreeding depression, disease, and human‐wildlife conflict (Bicca‐Marques et al. 2020). Despite their uncertain long‐term viability, individuals from these populations may represent valuable components of metapopulation‐based conservation strategies through translocation to more suitable habitats (see Greggor et al. 2024; IUCN/SSC 2013; Morris et al. 2021).

The success of translocation projects depends on the ability of released individuals to survive, establish, and reproduce in the recipient population (Greggor et al. 2024), processes that may be constrained by behavioral limitations (Berger‐Tal et al. 2020; Tetzlaff et al. 2019). These challenges underscore the importance of understanding how individuals respond to variations in habitat quality, as behavioral adjustments may determine both persistence in tolerated environments and the effectiveness of conservation interventions aimed at promoting long‐term population viability (Berger‐Tal and Blumstein 2024; Sievers et al. 2024). Consequently, it is important to assess whether the behavioral adjustments that facilitate persistence under suboptimal conditions (Sol et al. 2013; Wong and Candolin 2015) remain reversible or instead compromise the ability of translocated individuals to survive and reproduce after release in habitat patches capable of supporting populations in the long term (Greggor et al. 2024; Sievers et al. 2024).

Primates that survive in lower‐quality, disturbed habitats with restricted areas and lower richness and abundance of food species are behaviorally and dietarily flexible, often use small home ranges and venture into the matrix for dispersal, foraging, or refuge (Arroyo‐Rodríguez and Mandujano 2006; Estrada et al. 2012; Galán‐Acedo et al. 2019; Kalbitzer and Chapman 2018; Lino et al. 2019; Marsh 2003; Tutin and Lee 1999). These characteristics are found in many taxa (e.g., strepsirhines: *Eulemur rubriventer* and *Eulemur fulvus rufus*: Overdorff 1993; *Daubentonia madagascariensis*: Sterling and McCreless 2006; catarrhines: *Colobus vellerosus*: Wong et al. 2006; *Semnopithecus vetulus*: Dela 2011; platyrrhines: *Callithrix geoffroyi*: Passamani and Rylands 2000; *Alouatta* spp.: Bicca‐Marques et al. 2020; *Aotus lemurinus*: Bustamante‐Manrique et al. 2021).

Howler monkeys (*Alouatta* spp.) provide a useful model for investigating the behavioral responses to habitat patch size (Kowalewski et al. 2015b). They have the largest distribution range among the neotropical non‐human primates, occurring from Mexico to northern Argentina and southern Brazil, over an altitude gradient from sea level to 3200 m (Kowalewski et al. 2015b). They stand out among the platyrrhines for their tolerance to spatial habitat restriction (i.e., reduction in habitat patch size) and disturbed forest patches (Bicca‐Marques 2003; Bicca‐Marques et al. 2020; Di Fiore et al. 2011; Kowalewski et al. 2015a, 2015b). Their survival in small habitat patches, where the qualitative and quantitative availability of food plant resources is lower than in large patches or continuous forests (Arroyo‐Rodríguez and Dias 2010), has been linked primarily to the ability to subsist on a diet rich in leaves and diverse in terms of food sources and items (Bicca‐Marques 2003; Dias and Rangel‐Negrín 2015; McKinney 2019).

In addition to extensive field studies on some species, two genus‐wide literature reviews (Bicca‐Marques 2003; Fortes et al. 2015) and one *Alouatta palliata* review (Cristóbal‐Azkarate and Arroyo‐Rodríguez 2007) have assessed the influence of habitat patch size on aspects of the activity budget, diet composition, or ranging behavior. The *A. palliata* review also assessed the relationship of population density with the response variables. The two earlier reviews assessed the relationships between the predictor variable(s) and behavioral response variables using univariate statistical approaches, including correlation and regression analyses. The review by Fortes et al. (2015) focused on home range size and day range and employed a multivariate modeling framework that allowed the simultaneous evaluation of habitat patch size and other potential predictors.

Both earlier reviews found positive relationships between habitat patch size and home range size, irrespective of whether studies in which home range equaled habitat patch size were included (Bicca‐Marques 2003) or excluded (Cristóbal‐Azkarate and Arroyo‐Rodríguez 2007) from the analysis. The former also noted a concentration of observations at the lower end of the habitat patch size gradient, where many groups occupied habitat patches smaller than the home range typically used under less spatially constrained conditions. In contrast, habitat patch size did not predict home range size in the study by Fortes et al. (2015). Both Fortes et al. (2015) and Cristóbal‐Azkarate and Arroyo‐Rodríguez (2007) found a negative relationship between population density and home range size.

There were no significant relationships between habitat patch size and day range (Bicca‐Marques 2003; Fortes et al. 2015). However, Fortes et al. (2015) found that day range increased with group size and decreased with population density and the dietary contributions of both fruits and leaves.

The two studies assessing activity budgets found no significant relationships of time spent resting, moving, and feeding with habitat patch size, and the same pattern was true for the dietary contribution of both leaves and fruits (Bicca‐Marques 2003; Cristóbal‐Azkarate and Arroyo‐Rodríguez 2007). Finally, habitat patch size was positively related to diet richness (i.e., the number of food species) across the genus (Bicca‐Marques 2003). However, the opposite pattern was observed within *Alouatta palliata*, where habitat patch size was negatively related to diet richness, while population density showed a positive relationship with this variable (Cristóbal‐Azkarate and Arroyo‐Rodríguez 2007).

In this study, we aimed to identify the effects of habitat patch size (a proxy of habitat quality), group size, and the number of observation hours on howler monkey activity budget, diet composition, and use of space across a gradient ranging from small forest fragments to continuous forests. In addition to expanding the database with over a decade of literature published since the previous reviews, we aimed to identify relationships among the examined behavioral and ecological variables. Specifically, we hypothesized that home range size would increase with habitat patch size until reaching an asymptote corresponding to the species‐typical home range observed in large forest fragments and continuous forest (e.g., *A. seniculus*, 182 ha, Palacios and Rodriguez 2001), with home range constrained in patches smaller than that threshold (e.g., *A. caraya*, 0.7 ha, Prates and Bicca‐Marques 2008; see also Arroyo‐Rodríguez and Dias 2010). In contrast, we hypothesized that habitat patch size would not predict day range (rarely exceeding 1 km; Fortes et al. 2015), given the evolved energy‐conservation strategy characteristic of howlers, which facilitates the digestion of the higher‐fiber, lower‐energy leafy component of their diet (Milton 1998). Because larger groups require more food (Agostini et al. 2012; Fortes et al. 2015), we expected that day range would increase with group size (Fortes et al. 2015). However, this expectation may be moderated by habitat patch size, because preferred resources are likely to be more spatially concentrated in small patches than in large ones. Regarding the activity budget, we hypothesized that time spent feeding would not vary with habitat patch size (Bicca‐Marques 2003; Cristóbal‐Azkarate and Arroyo‐Rodríguez 2007). In contrast, we expected feeding time to increase with the contribution of leaves to the diet (unlike Fortes et al. 2015) and decrease with the contribution of fruit (Fortes et al. 2015; Stevenson et al. 2015), because fruit generally yields a higher biomass intake rate than leaves (Aristizabal et al. 2017; Fernández and Kowalewski 2018). In relation to diet composition, we hypothesized that the dietary contributions of both leaves and fruit would also not vary with habitat patch size, consistent with previous reviews (Bicca‐Marques 2003; Cristóbal‐Azkarate and Arroyo‐Rodríguez 2007). This pattern may reflect the availability of cultivated or non‐native fruits in orchards and the surrounding matrix (Bicca‐Marques and Calegaro‐Marques 1994; Chaves and Bicca‐Marques 2017; Dias and Rangel‐Negrín 2015), which could weaken the relationship between habitat patch size and fruit consumption at some study sites. However, an alternative expectation is that leaf consumption decreases and fruit consumption increases with habitat patch size or home range size (Juan et al. 2000; but see Marsh 1999), because fruit availability, typically greater in larger forest fragments, is more closely reflected in consumption patterns than is the availability of young leaves (Estrada et al. 1999; Milton 1980; Stevenson et al. 2015). Finally, the literature provides contrasting predictions regarding the influence of habitat patch size on diet richness. The genus‐wide review supports a positive relationship between diet richness and habitat patch size (Bicca‐Marques 2003), whereas the review focused on *A. paliatta* supports a negative one (Cristóbal‐Azkarate and Arroyo‐Rodríguez 2007). Although howler monkeys can subsist on a relatively narrow diet in species‐poor forest fragments by relying on abundant or asynchronous fruiting resources such as *Ficus* spp. (Bicca‐Marques 2003; Bolt et al. 2021; Chaves and Bicca‐Marques 2016; Cristóbal‐Azkarate and Arroyo‐Rodríguez 2007; Horwich 1998), they may also broaden their diet by incorporating cultivated non‐native species, lianas, and palms (Bicca‐Marques and Calegaro‐Marques 1994; Chaves and Bicca‐Marques 2016, 2017; Dias and Rangel‐Negrín 2015).

Understanding the relationships between habitat patch size and howler monkey behavior and ecology (Kowalewski et al. 2015b) is essential for assessing whether non‐self‐sustaining populations inhabiting small forest fragments can contribute to metapopulation persistence in fragmented landscapes that would otherwise function as tolerance niches. Translocations can aim either to reinforce existing populations by increasing population size and genetic diversity, or to reintroduce species to suitable habitats from which they were extirpated by causes that have since ceased, such as hunting and yellow fever outbreaks (Almeida et al. 2019; Crockett 1998; Gorostiaga et al. 2026; Moreno et al. 2015; Possamai et al. 2022), thereby re‐establishing ecological interspecific interactions and processes like seed dispersal and forest regeneration (Camaratta et al. 2017; Genes et al. 2018). Ultimately, this knowledge can also help improve habitat quality and connectivity in fragmented landscapes.

## 2 Methods

### 2.1 Ethics Statement

All studies included in this review are previously published works reporting non‐invasive, observational research on non‐human primates. As this study is based solely on the analysis of published literature, we did not independently verify compliance with institutional ethical approvals, legal requirements, or professional guidelines for each individual study. However, these studies were conducted under the standards and regulations in place at the time of data collection, which may have varied across periods and locations. We note that more recent studies explicitly report adherence to the Principles for the Ethical Treatment of Non‐Human Primates and, where applicable, the Code of Best Practices for Field Primatology of the American Society of Primatologists.

### 2.2 Compilation of Information

We based this research on an extensive literature review using keywords in English, Portuguese, and Spanish on the Web of Science and Google Scholar databases. The English keywords used were “*Alouatta*” OR “howler monkey” AND “diet*” OR “feed*” OR “home range*” OR “use of space” OR “daily path” OR “ranging” OR “distance traveled” OR “day range” OR “behav*” OR “ecol*.” We considered all documents reviewed by Bicca‐Marques (2003), Dias and Rangel‐Negrín (2015), and Fortes et al. (2015), and extended the review to encompass peer‐reviewed articles, book chapters, doctoral theses, and master's dissertations published from the early 2000s through November 2025 that addressed howler monkey (*Alouatta* spp.) behavior, diet, and space use. The studies newly added in this review are available online, unlike some older theses and dissertations used in Bicca‐Marques (2003) that are also included in our database. We report this review according to the PRISMA 2020 guidelines (Page et al. 2021; Figure S1).

**Figure 1:**
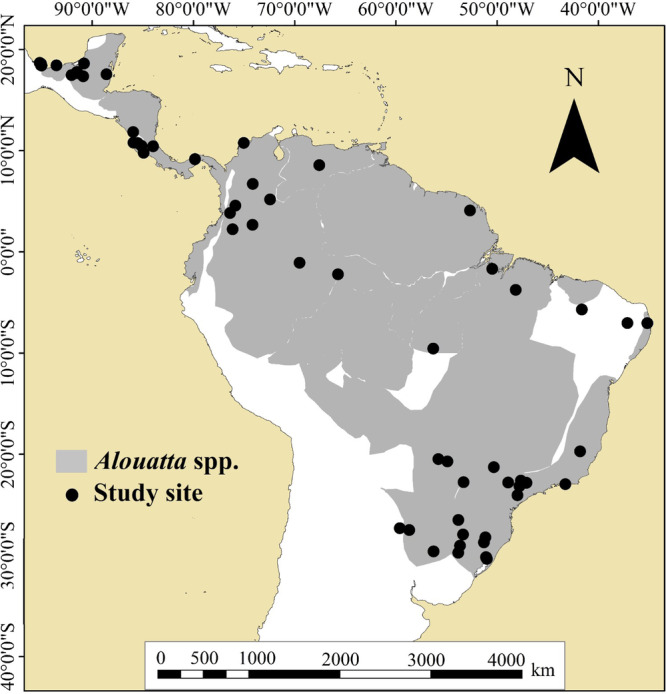
Geographic distribution of *Alouatta* spp. (gray area), and location of the studies included in this research (black circles).

We excluded studies that did not focus on howler monkeys or did not address the variables of interest. We further restricted the dataset to studies with at least 200 h of behavioral data collection per social group distributed over a minimum period of 8 months to reduce the risk of bias associated with limited observation time. Studies not meeting these criteria are listed in Table S1. Mean (±SD) observation time across studies was 619 ± 414 h (median = 502 h; range = 208–2357 h). From a total of 3320 non‐duplicated documents, we compiled data from 97 studies on 124 social groups representing nine species conducted at 62 locations (Figure 1; Table S2).

We recorded the habitat patch size (in ha) and the following information for each study group, whenever available: home range size (ha), day range (m), investment in resting and in feeding (% daytime), contribution of leaves in general, mature leaves, and young leaves to the diet (% of feeding records or feeding time for each item, calculated based on scan or focal‐animal data, respectively; hereinafter, leaf consumption, mature or young leaf consumption), contribution of fruits in general, ripe fruits, and immature fruits to the diet (calculated as described above for leaves; hereinafter, fruit consumption, ripe or immature fruit consumption), number of plant species used as food sources (hereinafter, diet richness), group size (number of individuals of all age classes), and observation time (h). We used observation time as a proxy for seasonal coverage, as studies with fewer observation hours tend to be more temporally clustered. We did not assess the investment in locomotion because it is often collinear with resting and/or feeding investment (Prates et al. 2018), and day range more reliably captures investment in group travel.

### 2.3 Data Processing

Before model fitting, we assessed multicollinearity among predictors in our multivariate models using the “*car*” package in R (Fox and Weisberg 2019), adopting a Variance Inflation Factor (VIF) < 4 as threshold for variable retention (Neter et al. 1996). We later excluded resting time from subsequent analyses to avoid analytical redundancy with feeding time and to optimize model stability, given its correlations with feeding time and diet richness (Pearson's *r* = –0.48 and –0.45, respectively), which increased VIF in global models. The remaining variables were habitat patch size, home range size, day range, feeding time, fruit and young leaf consumption, diet richness, group size, and observation time. Feeding time was analyzed solely as a response variable, as it is generally interpreted as a behavioral outcome of habitat restriction, dietary composition, and space use rather than as a causal predictor (Aristizabal et al. 2019; Cristóbal‐Azkarate and Arroyo‐Rodríguez 2007; Behie and Pavelka 2012; Chaves and Bicca‐Marques 2016). Habitat patch size, group size, and observation time entered only as predictor variables. The remaining variables were modeled both as response and predictor variables. We used Generalized Linear Mixed Models (GLMM, Knudson 2024) to identify potential predictors of the response variables using distributions appropriate to the data (Warton et al. 2016).

We identified extreme outliers using the “*qqplot*” function from the “*car*” package (Fox and Weisberg 2019) in R software version 4.5.1 (R Core Team 2026). We excluded the following outliers to avoid biasing the analyses: home range size (182, 111, and 108 ha), diet richness (195 species), and group size (59 and 40 individuals). All continuous predictors and response variables were Z‐transformed (scaled to a mean of 0 and a standard deviation of 1) prior to analysis to improve the interpretability of model coefficients and ensure comparability across predictors with different scales. We used the “Gaussian” family for all variables.

### 2.4 Statistical Modeling

We assessed the influence of biologically relevant candidate predictors and observation time (fixed effects) on six response variables. We designed models to assess whether and how: (a) habitat patch size, group size, and observation time influence home range; (b) habitat patch size, home range, and group size influence day range; (c) habitat patch size, home range, group size, fruit and young leaf consumption, and diet richness influence feeding time; (d–e) habitat patch size, home range, day range, group size, and diet richness influence young leaf consumption and fruit consumption, respectively; and (f) habitat patch size, home range, day range, group size, and observation time influence diet richness. All models included species identity and study sites as random effects to account for non‐independence in the data, with species controlling for interspecific differences and study site accounting for repeated sampling of the same location or multiple social groups.

Since most studies available only addressed a subset of the variables of interest, we used a two‐step analytical approach to prevent overfitting, minimize data loss, and optimize the sample size for the analyses of each response variable. First, we ran univariate mixed‐effects models for each predictor and compared their fit (AICc) against a null model containing only the intercept and random effects. Predictors that failed to outperform the null model were considered uninformative and excluded from subsequent steps. Second, we applied different criteria based on the univariate screening results for each response variable: (i) if no variable outperformed the null model, we concluded that the response variable was not predictable by any potential predictor; (ii) if only one variable outperformed the null model, we considered it a good predictor; and (iii) if at least two variables outperformed the null model, we ran a global model containing them. Third, we generated all possible global sub‐models using the “dredge” function in the “MuMIn” package in R version 4.5.1 (R Core Team 2026) to assess if they were good predictors.

Model selection and inference followed an information‐theoretic (IT) framework (Anderson 2008; Burnham and Anderson 2002; Burnham et al. 2011). We performed full model averaging across the entire set of candidate models to account for model selection uncertainty and avoid arbitrary ΔAICc thresholds. We assessed the influence of predictors based on the magnitude and direction of their standardized coefficients (*β*), unconditional standard errors (SE), and 95% confidence intervals (95% CI). We extracted these parameters from either the unique univariate model or the final averaged model. We interpreted effect sizes in terms of their magnitude and precision rather than statistical significance. For model‐averaged predictors, we also calculated their relative importance (*Σwi*) by summing the Akaike weights of all models that included them. Finally, we reported both marginal (*R^2^m*, variance explained by fixed effects) and conditional (*R^2^c*, variance explained by both fixed and random effects) coefficients of determination.

## 3 Results

Howler monkey groups spent, a mean (±SD, range) of 63.1% (±9.3%, 39.9%–84.7%) of their time resting (*n* = 78 groups) and 17.5% (±5.3%, 6.0%–29.0%) feeding (*n* = 80). Leaves accounted for a mean of 58.3% of feeding events (±14.6%, 13.0%–85.0%, *n* = 77; young leaves: 34.9% ± 15.2%, 6.5%–76.7%, *n* = 59; mature leaves: 21.7% ± 15.9%, 1.0%–75.3%, *n* = 59) and fruits, 29.8% (±14.0%, 2.0%–61.0%, *n* = 78; immature fruits: 7.6% ± 6.1%, 0.1%–21.0%, *n* = 35; ripe fruits: 24.7% ± 14.0%, 1.9%–58.0%, *n* = 35). A mean of 41 plant species (±17, 14–74 spp., *n* = 66) were used as food sources. Social groups (11 ± 6 individuals, 4–29 individuals, *n* = 116) used mean home ranges of 16.1 ha (±21.1, 0.2–92.0 ha, median = 9.2 ha, *n* = 95) within a wide range of habitat patch sizes (mean = 14,986 ± 113,022 ha, 0.3–1,240,000 ha, median = 127.5 ha, *n* = 124), where they covered a mean distance of 525 m per day (±208, 123–1150 m, *n *= 57).

**Figure 2:**
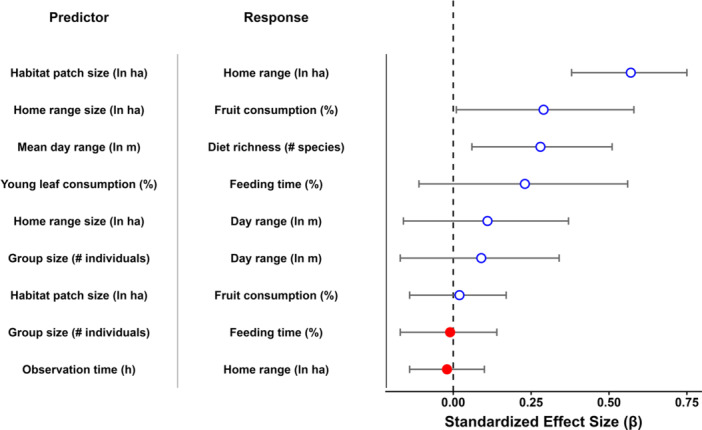
Standardized effect sizes of the relationships between predictor and response variables. Natural logarithms are denoted as “ln.” Open circles (blue outline) indicate mostly positive effects; solid circles (red filled) indicate mostly negative effects.

**Figure 3:**
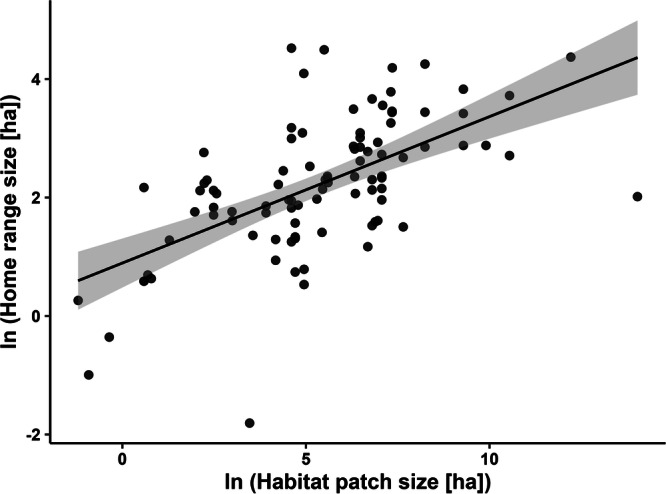
Relationship between habitat patch size and home range size in howler monkeys.

Habitat patch size was a strong predictor with a positive effect on home range size (*∑wi* = 1.00, *β* = 0.56; Figures 2 and 3). Observation time, despite marginally outperforming the null model in the univariate screening (Table S3), had its predictive power lowered when analyzed together with patch size (*∑wi* = 0.31). Also, the 95% confidence interval of its standardized effect size crossed zero (Figure 2). The fixed effects (*R^2^m*) explained 31% of the variance in home range size, whereas the full model (*R^2^c*) explained 52% of it (Table 1).

**Table 1:** Summary of model results for howler monkey behavioral variables.

Response variables	Predictor variables	*β*	SE	95% CI	Importance (*Σwi*)	*n*
Home range (log ha)	Habitat patch size	0.56	0.10	[**0.37, 0.76**]	1.00	95
(*R^2^m* = 0.31, *R^2^c* = 0.52)	Observation time	−0.03	0.07	[−0.16, 0.11]	0.31	95
Day range (log m)	Home range	0.11	0.14	[−0.16, 0.37]	0.52	53
(*R^2^m* = 0.08, *R^2^c* = 0.55)	Group size	0.09	0.13	[−0.17, 0.34]	0.45	53
Feeding time (%)	Young leaf consumption	0.22	0.17	[−0.11, 0.56]	0.77	48
(*R^2^m* = 0.12, *R^2^c* = 0.53)	Group size	−0.01	0.08	[−0.17, 0.14]	0.23	48
Fruit consumption (%)	Home range	0.29	0.14	[**0.01, 0.58**]	0.91	61
(*R^2^m* = 0.15, *R^2^c* = 0.48)	Habitat patch size	0.02	0.08	[−0.14, 0.17]	0.26	61
Diet richness (nr. species)	Day range	0.28	0.12	[**0.06, 0.51**]	—	39
(*R^2^m* = 0.10, *R^2^c* = 0.84)						

*Note:* Parameters: Standardized model‐averaged coefficient (*β*); Unconditional standard error (SE); 95% confidence interval (95% CI, in bold when not crossing the zero); Sum of Akaike weights representing the relative importance of the variable (*Σwi*); n (sample size used in the multivariate or univariate model). *R^2^m* and *R^2^c* are the marginal and conditional coefficients of determination for the global model, respectively.

Day range was not predicted by any of the evaluated variables. After the exclusion of habitat patch size in the univariate screening (Table S4), home range size and group size showed moderate relative importance in the multivariable model (*∑wi* = 0.52 and 0.45, respectively), but the 95% CIs of their standardized effect sizes crossed zero (Figure 2). The fixed effects explained only 8% of the variance in day range, whereas the full model explained 55% of it (Table 1).

Feeding time was not predicted by any of the evaluated variables. Only young leaf consumption and group size outperformed the null model (Table S5) and were retained for the multivariable analysis. Young leaf consumption showed the highest relative importance, whereas group size showed low importance (*∑wi* = 0.77 and 0.23, respectively). The 95% CI of their standardized effect sizes crossed zero (Figure 2). The fixed effects explained 12% of the variance in feeding time, whereas the full model explained 53% of it (Table 1).

Young leaf consumption was not predicted by any of the analyzed variables. None of them outperformed the null model in the univariate screening (Table S6).

**Figure 4:**
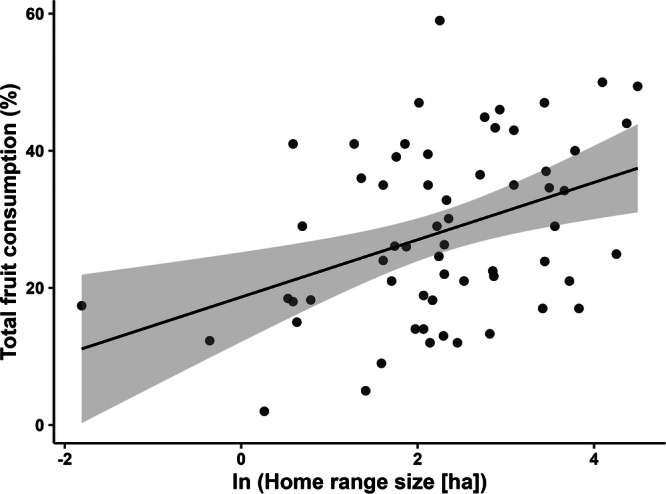
Relationship between home range size and consumption of fruits by howler monkeys.

Only habitat patch size and home range size outperformed the null model in the analyses of fruit consumption (Table S7) and were retained for the multivariable analysis. Home range size was the strongest predictor with a positive effect on fruit consumption (*∑wi* = 0.91, *β* = 0.29; Figures 2 and 4). Habitat patch size had weak predictive power (*∑wi* = 0.26) and the 95% confidence interval of its standardized effect size crossed zero (Figure 2). The fixed effects explained 15% of the variance in fruit consumption, while the full model explained 48% of it (Table 1).

**Figure 5:**
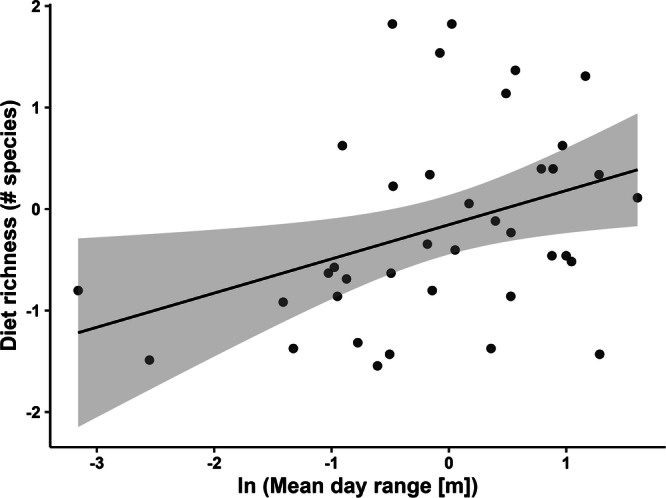
Relationship between day range and diet richness in howler monkeys.

Finally, day range was the only predictor of diet richness to outperform the null model during the univariate screening (Table S8). It showed a positive effect on diet richness (*β* = 0.28; Figures 2 and 5). While day range explained 10% of the variance in diet richness, the full model explained 84% of it (Table 1).

## 4 Discussion

We assessed whether habitat patch size and group size modulate several aspects of howler monkey behavior and ecology based on a thorough review of studies carried out across the genus's distribution. We also assessed the relationships between the analyzed variables and whether study duration predict home range size and diet richness. Habitat patch size predicted home range size, which influenced fruit consumption. Additionally, day range influenced diet richness.

The positive association between habitat patch size and home range size was expected given that howler monkeys can occupy patches much smaller than the home ranges typically observed in larger forests (Bicca‐Marques 2003; Cristóbal‐Azkarate and Arroyo‐Rodríguez 2007). Differences in resource distribution, quality, and availability between habitat patches (Chaves and Bicca‐Marques 2016; Chaves‐Diaz et al. 2026; Prates and Bicca‐Marques 2011; van Schaik et al. 1993), the presence and density of interspecific competitors, and differences in population density also modulate home range size (Fortes et al. 2015).

As also expected according to previous reviews (Bicca‐Marques 2003; Fortes et al. 2015), habitat patch size did not predict day range. This result aligns with the strategy of conserving energy for food digestion (Milton 1998), as reflected in the relatively short day ranges, which average around 500–600 m and rarely exceed 1000 m (Fortes et al. 2015). The weak relationship between group size and day range, contrary to the findings of Fortes et al. (2015), likely reflects variation in habitat quality among patches that is independent of patch size. This interpretation is supported by the substantially greater proportion of variance explained by the random effects, indicating that differences among ecological contexts contributed more to variation in day range than the measured predictors. Between‐site differences in the availability and spatial distribution of resources (Camaratta et al. 2017; Ceccarelli et al. 2019; Hopkins 2011; Raño et al. 2016; Rivillas‐Carmona et al. 2026) may have contributed to the lack of an effect of the degree of frugivory and folivory on day range, contrary to the negative relationships found by Fortes et al. (2015). For example, howler monkeys may “camp” in areas with high concentrations of fruiting trees (Fortes et al. 2015; Palacios and Rodriguez 2001), thereby reducing travel requirements even when fruit consumption is high.

The predominantly positive but uncertain effect of young leaf consumption on feeding time is consistent with the lower intake rate of leaf biomass per unit time relative to fruit biomass (Aristizabal et al. 2017; Fernández and Kowalewski 2018). Variation in feeding patch size and productivity (Hopkins 2016) affect the predictive powers of both young leaf consumption and group size. Because resting time was not modeled due to its collinearity with feeding time, and no variable predicted day range (a proxy for moving time), any effect of habitat patch size on resting time remains unclear, although we found no indirect support for such an effect. This interpretation is consistent with the findings of reviews examining the effects of habitat patch size on activity budgets (Bicca‐Marques 2003; Cristóbal‐Azkarate and Arroyo‐Rodríguez 2007).

The lack of good predictors of young leaf consumption among the tested variables corroborates studies that have assessed the effects of habitat patch size at both the genus and *Alouatta palliata* levels (Bicca‐Marques 2003; Chaves and Bicca‐Marques 2013; Cristóbal‐Azkarate and Arroyo‐Rodríguez 2007). Unlike the consumption of young leaves, fruit consumption increased with home range size, likely reflecting greater fruit availability in larger habitat patches and lower availability in small or degraded forest fragments (Faria et al. 2023; Fortes et al. 2015). The imprecision of the effect size estimates and the low explanatory power of home range and habitat patch size are consistent with high variability in fruit availability among habitat patches (Behie and Pavelka 2015). For example, many small forest fragments where howler monkeys have been studied are either adjacent to orchards of non‐native fruit species or embedded in landscapes with nearby cultivated orchards (Bicca‐Marques and Calegaro‐Marques 1994; Chaves and Bicca‐Marques 2017; Pozo‐Montuy et al. 2013; Prates and Bicca‐Marques 2008). It is likely that the exploitation of lianas, palms, and cultivated non‐native species, which may be more abundant in smaller forests (Bicca‐Marques and Calegaro‐Marques 1994; Chaves and Bicca‐Marques 2016; Dias and Rangel‐Negrín 2015), or the consumption of resources from a greater number of plant families in specific areas of smaller habitats (e.g., forest edges; Bolt et al. 2021), compensate for the reduced availability of native or preferred foods in small and disturbed forests (Chaves and Bicca‐Marques 2016, 2017).

The unexpected absence of a relationship between habitat patch size and diet richness may reflect differences in plant community composition among study sites. This interpretation is consistent with diet richness exhibiting the greatest proportion of variance attributable to the random effects among all response variables analyzed. For example, the availability of fleshy fruit of cultivated non‐native species within or adjacent to small habitat patches at several study sites may have reduced differences in diet richness among habitat patches varying in plant diversity by supplementing food resources in less diverse patches (e.g., Chaves and Bicca‐Marques 2016). Although day range explained only a small fraction of the variation in diet richness, the increase in the number of plant species exploited as food sources with increasing mean day range is compatible with the goal of nutrient mixing (Raubenheimer and Simpson 2004; Westoby 1974) in howler monkey foraging (Righini et al. 2020; see, also, Garber and Kowalewski 2015). This trend would likely persist if the analysis distinguished between reproductive and vegetative plant parts within the same food species.

Although longer behavioral sampling is expected to increase the likelihood of recording larger home ranges and the consumption of temporally restricted or low‐density food items, the effect of observation time on home range size was uncertain. Additionally, observation time accounted for less than 1% of the variation in diet richness. These findings are consistent with our decision to restrict the literature review to studies with at least 200 h of data collection conducted over 8 or more months.

The limited and uncertain predictive power of habitat patch size for variables other than home range size is consistent with the view that behavioral flexibility can facilitate persistence under heterogeneous environmental conditions (Sih 2024). However, three alternative, non‐mutually exclusive interpretations are possible given the predominance of null effects among the study variables, the modest effect sizes of the three relationships showing clear directionality, and the substantially higher conditional than marginal *R^2^* values that indicate that a greater proportion of the variance was associated with random effects (e.g., other characteristics of habitat patches, their inhabitants, and the surrounding landscape). Models including other predictors also showed a greater proportion of variance associated with random effects. The first alternative interpretation is insufficient statistical power potentially related to modest sample sizes within some study species and high ecological heterogeneity across studies. Second and also related to ecological heterogeneity, habitat patch size may be a poor proxy for habitat quality. This interpretation is compatible with differences between study sites in floristic composition, climate, food availability, quality, and spatial distribution, proximity to sources of food supplementation in the matrix, population density, and social group structure, along with the composition of competitor, predator, and parasite guilds. These selective pressures interact to shape howler monkey behavior and ecology. Between‐site differences in ecological conditions have been suggested to explain intra‐ and interspecific differences in the contribution of fruits to the diet of howler monkeys (Behie and Pavelka 2015). Third, the heterogeneity across species and, especially, ecological contexts throughout the distribution of *Alouatta* spp. may also contribute to the absence of consistent patterns across the genus. In short, we corroborate previous evidence that howler monkeys can survive in forested areas of varying sizes without departing from their typical behavioral pattern, which is dominated by resting and a folivorous–frugivorous diet (Bicca‐Marques 2003; Di Fiore et al. 2011; Dias and Rangel‐Negrín 2015).

Although howler monkey populations are unlikely to persist long term in small, isolated habitat patches (Bicca‐Marques et al. 2020), their behavioral and ecological flexibility suggests that individuals in these tolerance niches may contribute to conservation strategies in fragmented landscapes (Bay‐Jouliá et al. 2026; McKinney 2019; Repullés and Galán‐Acedo 2025). The greater similarity in diet composition among geographically closer study groups (Chaves and Bicca‐Marques 2013) raises the possibility that translocations within the same microregion may facilitate adjustment to novel environments. Likewise, given the importance of learning for adaptation to environmental novelty (Peterson et al. 2025; Sol et al. 2013), soft‐release protocols that promote familiarization with release sites may improve survival and site fidelity (Resende et al. 2021; Tetzlaff et al. 2019). Small habitat patches may also function as stepping stones (Bicca‐Marques and Calegaro‐Marques 1994), facilitating gene flow, providing refuges during environmental catastrophes, or harboring individuals with traits that enhance tolerance to pathogens. Collectively, these considerations are compatible with conserving forest fragments and their howler monkey populations, and integrating them into metapopulation‐based management, may enhance connectivity and reduce the risk of regional extirpation in human‐modified landscapes.

## Author Contributions

**Sebastián Bustamante‐Manrique:** conceptualization, investigation, writing – original draft, methodology, validation, visualization, writing – review and editing, formal analysis, project administration, data curation. **Vinícius Klain:** investigation, methodology, validation, visualization, writing – review and editing, formal analysis, data curation. **Júlio César Bicca‐Marques:** conceptualization, writing – original draft, methodology, visualization, writing – review and editing, supervision, resources, validation, funding acquisition, project administration.

## Supporting information


**Supporting File 1:** ajp70182‐sup‐0001‐Figure_S1_PRISMA_fluxogram.

**Supporting File 2:** ajp70182‐sup‐0002‐Table_S1_Depuration_database.

**Supporting File 3:** ajp70182‐sup‐0003‐Table_S2_Howler_monkey_database_Bustamante_Manrique_et_al_2026.

**Supporting File 4:** ajp70182‐sup‐0004‐Table_S3_Home_Range.

**Supporting File 5:** ajp70182‐sup‐0005‐Table_S4_Day_Range.

**Supporting File 6:** ajp70182‐sup‐0006‐Table_S5_Feeding_Investment.

**Supporting File 7:** ajp70182‐sup‐0007‐Table_S6_Young_Leaves.

**Supporting File 8:** ajp70182‐sup‐0008‐Table_S7_Fruit_Consumption.

**Supporting File 9:** ajp70182‐sup‐0009‐Table_S8_Diet_Richness.

## Data Availability

The data that supports the findings of this study are available in the Supporting material of this article.
