# Supplementary material for: Behavioral Flexibility and the Conservation Value of Howler Monkey Populations in Small Habitat Patches
Source: Am J Primatol. 2026 Jun 23;88(6):e70182. doi: 10.1002/ajp.70182 (PMC13288005; doi:10.1002/ajp.70182)
Supplement: Supplementary file 1 — Supporting File 1: ajp70182‐sup‐0001‐Figure_S1_PRISMA_fluxogram. [file AJP-88-e70182-s001.docx]

**Supplementary information**

**Behavioral flexibility and the conservation value of howler monkey populations in small habitat patches**

Sebastián Bustamante-Manrique^1,2^, Vinícius Klain^1,3^ & Júlio César Bicca-Marques^1^

^1^ Laboratório de Primatologia, Escola de Ciências da Saúde e da Vida,
Pontifícia Universidade Católica do Rio Grande do Sul, PUCRS, Porto Alegre, Brazil

^2^ Programa de Pós-Graduação em Ecologia e Conservação da Biodiversidade,
Laboratório de Ecologia Aplicada à Conservação,
Universidade Estadual de Santa Cruz, UESC, Ilhéus, Brazil

^3^ Centro de Biotecnologia, Departamento de Biologia Molecular e Biotecnologia,
Universidade Federal do Rio Grande do Sul, UFRGS, Porto Alegre, Brazil

**Corresponding author:**

Júlio César Bicca-Marques, Laboratório de Primatologia, Pontifícia Universidade Católica do Rio Grande do Sul, Porto Alegre, RS 90619–900, Brazil, E-mail: [jcbicca@pucrs.br](mailto:jcbicca@pucrs.br) , Phone: +55 (51) 3353-4742.

**Table S1.** PRISMA guidelines

**Search query in databases "Alouatta" OR "howler monkey" AND "diet*" OR "feed*" OR "home range*" OR "use of space" OR "daily path" OR "ranging" OR "distance traveled" OR "day range" OR "behav*" OR "ecol***

**Search query in databases "Alouatta" OR "howler monkey" AND "diet*" OR "feed*" OR "home range*" OR "use of space" OR "daily path" OR "ranging" OR "distance traveled" OR "day range" OR "behav*" OR "ecol***

Records identified through database searching

(*n* = 3,356)

- ISI Web of Science: 624

- Google Scholar: 1782

- Scopus: 806

- Dias & Rangel-Negrín, 2015; Fortes et al., 2015: 116

- Bicca-Marques, 2003: 28

Records excluded (*n* = 3,050)

- Duplicates: 62

- Records excluded by title/abstract: 2,988

(reasons: irrelevant to *Alouatta* ecology, use of space, behavior, or diet)

**Identification**

Records excluded (*n* = 209)

- Reasons: Raw data unavailable, less than 200 h of behavioral sampling, study covering less than 8 months, study in captivity, supplemented groups, translocated individuals, only immature individuals,congress

Records available for screening (*n* = 306)

**Screening**

Records included in analysis

(*n* = 97)

**Included**
